# Use of pH reagent strips to verify gastric tube placement in newborns*

**DOI:** 10.1590/1518-8345.3150.3227

**Published:** 2019-12-05

**Authors:** Flávia de Souza Barbosa Dias, Beatriz Pera de Almeida, Beatriz Regina Alvares, Rodrigo Menezes Jales, Jamil Pedro de Siqueira Caldas, Elenice Valentim Carmona

**Affiliations:** 1 Autonomous researcher, Valinhos, SP, Brazil.; 2 Universidade Estadual de Campinas, Hospital da Mulher Prof. Dr. José Aristodemo Pinotti, Campinas, SP, Brazil.; 3 Universidade Estadual de Campinas, Faculdade de Ciências Médicas, Campinas, SP, Brazil.; 4 Universidade Estadual de Campinas, Faculdade de Enfermagem, Campinas, SP, Brazil.

**Keywords:** Intubation, Gastrointestinal, Enteral Nutrition, Infant, Newborn, Hydrogen-ion Concentration, Gastric Juice, Neonatal Nursing, Intubação Gastrointestinal, Nutrição Enteral, Recém-Nascido, Concentração de Íons de Hidrogênio, Suco Gástrico, Enfermagem Neonatal, Intubación Gastrointerstinal, Nutrición Enteral, Recién Nascido, Concentración de Iones de Hidrógeno, Jugo Gástrico, Enfermería Neonatal

## Abstract

**Objective:**

to confirm the accuracy of the pH test in identifying the placement of the gastric tube in newborns.

**Method:**

double-blind, diagnostic test study conducted with 162 newborns admitted to a neonatal intensive care unit and an intermediate care unit. The subjects were submitted to enteral intubation, followed by pH test with reagent strip, which was analyzed by a nurse, and radiological examination, analyzed by radiologist. Blinding was kept among professionals regarding test results. Diagnostic accuracy analysis of the pH test in relation to the radiological exam was performed.

**Results:**

the sample consisted of 56.17% boys, with average birth weight of 1,886.79g (SD 743,41), 32.92 (SD 2.99) weeks of gestational age and the mean pH was 3.36 (SD 1.27). Considering the cutoff point of pH≤5.5, the sensitivity was 96.25%, specificity 50%, positive predictive value 99.35% and negative predictive value 14.29%.

**Conclusion:**

The pH test performed with reagent strips is sensitive to identify the correct placement of the gastric tube, so it can be used as an adjuvant technique in the evaluation of the gastric tube placement. In interpreting the results, pH ≤5.5 points to correct placement and values > 5.5 require radiological confirmation.

## Abstract (pt)

**Objetivo:**

confirmar a acurácia do teste de pH em identificar o posicionamento da sonda gástrica em recém-nascidos.

**Método:**

estudo de teste diagnóstico, duplo cego realizado com 162 recém-nascidos internados em unidade neonatal de terapia intensiva e unidade de cuidados intermediários. Os sujeitos foram submetidos à sondagem gástrica, em seguida realizado teste de pH com tira reagentes, que foi analisado por um enfermeiro, e exame radiológico, analisado por radiologista. Foi mantido o cegamento entre os profissionais com relação ao resultado dos testes. Realizada análise de acurácia diagnóstica do teste de pH em relação ao exame radiológico.

**Resultados:**

a amostra foi composta 56,17% por meninos, com média de peso de nascimento de 1.886,79g (dp 743,41), 32,92 (dp 2,99) semanas de idade gestacional e a média do pH foi 3,36 (dp 1,27). Considerando o ponto de corte de pH≤5,5, a sensibilidade foi 96,25%, especificidade 50%, valor preditivo positivo 99,35% e valor preditivo negativo 14,29%.

**Conclusão:**

o teste de pH realizado com tiras reagentes é sensível para identificar o posicionamento correto da sonda gástrica, assim pode ser utilizado como técnica adjuvante na avaliação do posicionamento da sonda gástrica. Na interpretação dos resultados, pH ≤5,5 indica posicionamento correto e valores >5,5 necessitam de confirmação radiológica.

## Abstract (es)

**Objetivo:**

confirmar la precisión del prueba de pH en identificar el posicionamiento de la sonda gástrica en recién nacidos.

**Método:**

estudio de prueba diagnóstico, doble ciego, realizado con 162 recién nacidos, internados en unidad neonatal de terapia intensiva y unidad de cuidados intermedios. Los sujetos fueron sometidos a sondaje gástrico, en seguida se realizó el prueba de pH con tira reactivas, el que fue analizado por enfermero y examen radiológico por especialista. Se manutuvo la ceguera entre los profesionales con relación al resultado de las pruebas. Se realizó un análisis de precisión diagnóstica del prueba de pH, en relación al examen radiológico.

**Resultados:**

la muestra estuvo compuesta 56,17% por niños, con media de peso de nacimiento de 1.886,79g (de 743,41), 32,92 (de 2,99) semanas de edad gestacional y la media del pH fue 3,36 (de 1,27). Considerando el punto de corte de pH≤5,5, la sensibilidad fue 96,25%, especificidad 50%, valor predictivo positivo 99,35% y valor predictivo negativo 14,29%.

**Conclusión:**

el prueba de pH realizado con tiras reactivas es sensible para identificar el posicionamiento correcto de la sonda gástrica, de ese modo puede ser utilizado como técnica adyuvante en la evaluación del posicionamiento de la sonda gástrica. En la interpretación de los resultados, un pH ≤5,5 indica posicionamiento correcto y valores >5,5 necesitan de confirmación radiológica.

## Introduction

Incorrect enteral gastric tube placement in newborns is associated with serious harms, including risk of death^(^ 1 ^-^ 2 ^)^. To confirm the placement of the tube immediately after its introduction and before each use is central to ensure the safe use of this device, so commonly used in neonatal inpatient units^(^ 3 ^-^ 6 ^)^.

The choice of the measurement method on tube insertion length is also part of the first precautions related to the insertion of this device. Currently, methods that use anatomical references are described in the literature, as well as equations that use the newborn’s weight or height to calculate insertion length. The most cited and recommended method is NEMU (nose, earlobe, mid-umbilicus), which is the distance from the tip of the nose to the earlobe and the midpoint between the xiphoid appendix and the umbilical base. In addition to this, we also found the ARHB (age-related, height based) and weight-based methods that use newborn height and weight respectively to calculate tube insertion length ^(^ 5 ^-^ 6 ^)^.

To evaluate the placement of the tube after insertion, the chest and abdomen radiological image is still considered the gold standard, since it allows the visualization of the entire course and the location of its distal extremity^(^ 7 ^-^ 9 ^)^. Despite being 100% accurate in the evaluation, it is not a suitable method to be routinely used in neonatal patients, due to the risks related to cumulative ionizing radiation exposure, besides the costs and timing that involve this procedure^(^ 4 ^,^ 10 ^)^.

A prevalence study conducted in 63 US hospitals showed that the methods that have been used in pediatric and neonatal clinical practice are, in order of options, checking the presence of residue on tube aspiration, auscultation of the epigastric region, verifying the external tube length, aspirated secretion pH testing, radiological examination and electromagnetic tracing^(^ 11 ^)^.

Regarding the accuracy and safety in the use of these alternative methods to radiological examination, it is known that auscultation of the epigastric region is not reliable and its discontinuity has been advised^(^ 10 ^,^ 12 ^)^. The presence of secretion to the aspiration of the tube and the evaluation of its color, as well as its appearance, may be sensitive in confirming the tube placement, but without established specificity and it is a confounding factor that endotracheal and bronchial secretions also may have the same color and appearance of gastric secretion^(^ 13 ^)^. Checking the outer length of the tube is recommended as an adjunctive measure in tube maintenance, but is not alone, because the distal end of the tube may move to the intestine or respiratory system even though the external fixation remains intact^(^ 5 ^)^. 

The electromagnetic tracing, despite presenting good consonance with the radiological examination, has an important limitation related to the tube bore, which should be at least 8Fr, which makes it impracticable to use this method to verify the placement of the tube in newborns^(^ 11 ^,^ 14 ^)^.

Verifying the pH of the aspirated secretion using reagent strips is a quick bedside test. Currently, there is a consensus among experts that this is the safest method available and is recommended as the first choice when verifying gastric tube placement in adults and children^(^ 9 ^-^ 10 ^,^ 12 ^)^.

The chain of gastric secretion production is complex and the main physiological stimulus for such production is feeding. It is known that stomach full development during the fetal period is up to the 14^th^ or 15^th^ gestational week and that 27-week-old preterm infants are capable of gastric pH <4.0 on the first day of life. However, in the first 48 hours after birth, the literature indicates that the pH may be higher due to low gastric acid secretion, lack of food or the presence of amniotic fluid in the stomach^(^ 15 ^-^ 17 ^)^.

In order to validate the current recommendation, this study aimed to confirm whether gastric pH values ​​≤5.5, found in newborn gastric tube aspirate is a sensitive and specific method for assessing the correct placement of the tube in the stomach. Moreover, as secondary objectives, to verify if the pH of the gastric aspirate was influenced by the diet, use of histamine H_2_ receptor antagonist drug and by the age of the studied sample.

## Method

This is a cross-sectional double-blind diagnostic test study conducted in a neonatal intensive care unit and intermediate care in a public teaching hospital. From October 2016 to July 2017, newborns who met the following inclusion criteria were selected: need for gastric tube for feeding; spontaneous breathing, without oxygen; absence of congenital malformations or syndromes; absence of surgical procedure in the digestive system. Exclusion criteria were considered to be under minimal manipulation care or to have nasogastric tube contraindication.

This study comes from a secondary analysis of a randomized controlled trial (REBEC Registry RBR-2zk6yc) aimed at verifying the difference in the correct placement of the gastric tube when using two different methods for measuring insertion length. For this purpose, a sample size of 162 subjects was calculated using the chi-square estimation methodology after a pilot study of 50 subjects, assuming a power of 80%, a significance level of 5% and a loss rate of 20%.

For this study, the power of the test was subsequently calculated to verify whether the sample size collected in the primary study would be adequate to test the hypothesis of the objective proposed here. The power of the test for sensitivity was 100% and specificity 25%, assuming a null value of 0.50 for sensitivity and specificity.

Each subject was included in the study on the first day of enteral feeding or on the day of changing the gastric tube, which occurred every 48 hours if the patient was initially using polyvinyl chloride (PVC) tube, according to the institution’s protocol where the data was collected.

For the present study we used pediatric enteral tubes size 6.5 (FrekaPaed^®^, Fresenius-Kabi^®^, Friedberg, Germany), inserted nasal route by one of the three research assistant nurses, who had at least 3 years of clinical experience. Two minutes before the procedure, up to 1ml of 25% sucrose was administered orally to alleviate the discomfort generated by the procedure^(^ 18 ^)^. The newborns were clinically stable, were not under minimal manipulation protocol and remained in easy retention during the procedure.

To estimate the tube insertion length, the NEMU (nose, earlobe, mid-umbilicus) measurement^(^ 19 ^)^ or the weight-based formula method^(^ 20 ^)^ were used. For the NEMU method, the distance between the tip of the nose and the ear lobe insertion point was verified, plus the distance between the ear lobe insertion point and the midpoint between the xiphoid appendix and the umbilical base. For the weight-based formula, the insertion length was estimated from the following calculation: 3x[weight in kilos]+13cm^(^ 20 ^)^.

**Figure 1:**
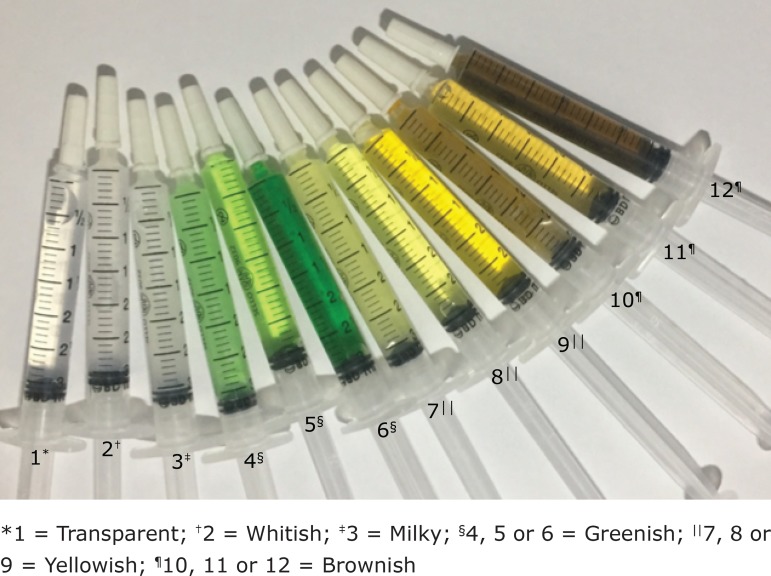
Color model for color evaluation of aspirated secretion ^*^1 = Transparent; ^†^2 = Whitish; ^‡^3 = Milky; ^§^4, 5 or 6 = Greenish; ^||^7, 8 or 9 = Yellowish; ^¶^10, 11 or 12 = Brownish

After insertion and fixation of the tube, a 3ml syringe was connected to the tube adapter and mild negative pressure was observed, with a return of secretion. Volume in ml and secretion staining were recorded according to a color model (Figure 1), used to standardize staining identification. For the elaboration of the color model, 3ml syringes and white, yellow, green and brown food coloring were used in different dilutions. The colors remained stable throughout the data collection.

Then the pH test was performed with one to two drops of the aspirated secretion on pH reagent strips, with scale from 2.0 to 9.0 and indicator every 0.5 point (MColorpHast^®^, Merk, Germany). PH values ​​≤5.5 were considered as indicative of gastric content and the tube classified as correctly placed as recommended by the UK National Patient Safety Agency^(^ 10 ^)^.

In case it was not possible to obtain secretion return with the aspiration of the tube, the patient was placed in the left lateral decubitus position and, after 15 minutes, a new attempt was made. A maximum of three attempts were required in each patient.

Radiological examination was performed in all individuals to confirm the placement of the tube. Chest and abdomen images were obtained in the supine position and with anteroposterior incidence. Radiological examinations were analyzed by a radiologist with experience in neonatal radiology, who was blinded to the pH test result, just as the research assistant nurses who performed the pH test were blinded to the radiological test result.

For analysis of the radiological image, it was considered as correct placement when the tip of the tube was observed inside the gastric bubble and could be located at the bottom, body or antrum. Placement was considered incorrect when the tip of the tube was seen above the gastroesophageal junction, in the cardia, pylorus or duodenal portion.

The sample was characterized according to gestational age at birth, corrected gestational age, birth weight, days of life, gender and diagnosis of hospitalization. Data were also collected on the type of feeding the newborn received prior to the tube procedure or if he/she was fasting, as well as whether or not he/she was receiving histamine H_2_-receptor antagonist.

Continuous variables are presented in terms of mean, standard deviation, median and minimum and maximum values. Categorical variables were expressed as absolute and relative frequency. For analysis of association and correlation between variables, Fisher’s exact test and Spearman’s correlation test were used respectively. For the accuracy analysis, the measures of prevalence, sensitivity, specificity, positive predictive value and negative predictive value were calculated^(^ 21 ^)^. All data collected were entered into a spreadsheet (Microsoft Excel for Mac, version 15.25) and analyzed using Statistical Analysis System (SAS) version 9.4. For all analyzes, a significance level of 5% was considered.

The study was part of a research project that was approved by the Ethics Committee under opinion number 1,376,279, following all recommendations of National Health Council Resolution 466/2012. The parents of the participating newborns voluntarily allowed inclusion of their children in the study by signing the informed consent form.

## Results

The study included 162 newborns, 91 of them male (56.17%). On average, they presented 32.92 (SD 2.99) weeks of gestational age (minimum 26.71; maximum 40.86) and 1886.79g (SD 743.41) of birth weight (minimum 750; maximum 4.160). Inclusion in the study occurred on average with 10.84 (SD 16.68) days of life (minimum 0; maximum 101) and 34.36 (SD 2.51) weeks of corrected gestational age (minimum 28.42; maximum 42). The most frequent diagnosis of hospitalization was prematurity (n=152; 93.83%), then respiratory disorders (n=122; 75.31%), metabolic disorders (n=39; 24.07%), infections (n=39; 24.07%), cardiocirculatory disorders (n=5; 3.09%) and others (n=21; 12.96%). No subjects were excluded from the sample.

Regarding to diet, it was observed that 106 newborns (65.43%) had received artificial milk or mixed feeding (breast milk and formula), 47 (29.01%) received pasteurized human milk or raw breast milk, while nine newborns (5.56%) were fasting before the procedure. For newborn infants who were being fed, the tube placement was performed one to two hours after receiving milk by gavage.

It was possible to obtain aspirated material from the tube in the first attempt in 145 cases (89.51%), in 12 cases (7.41%) in the second attempt and in five cases (3.08%) in the third attempt. Predominantly, the secretion color was whitish in 52.47% of the situations (n​=85), milky in 15.43% (n=25), transparent in 15.43% (n=25), greenish secretion in 7 , 41% of the sample (n=12), yellowish in 5.56% (n=9) and brownish in 3.70% (n=6). No association was found between secretion staining and tube placement.

The result of the aspirated secretion pH test was 3.36 (SD1.27; minimum 2.0; maximum 8.5). When classified according to the cutoff point established as a criterion in the evaluation of gastric tube positioning, 155 subjects (95.68%) had a pH ≤5.5 and seven had a result > 5.5.

There was use of gastric secretion inhibitor drug in only six subjects of the sample (3.70%) and in these cases the pH value was on average 6.16 (SD 1.94; minimum 3.0; maximum 8.5). Association between histamine H_2_ receptor antagonist use and pH> 5.5 was observed. In contrast, there was no relationship with the use of this drug and the difficulty in obtaining secretion in the first attempt to aspirate the tube (Table 1).

**Table 1:** Association between pH value of aspirated secretion, number of attempts to obtain secretion in tube aspiration and use of histamine H_2_ receptor antagonist (n=162). Campinas, SP, Brazil, 2017

Variable	H_2_ receptor antagonist				p-value*
	Yes		No		
	n	%	n	%	
PH value					<0.0001
>5.5	4	66.67	3	1.92	
≤5.5	2	33.33	153	98.08	
Attempts					0.1210
1 attempt	4	66.67	141	90.38	
2-3 attempts	2	33.33	15	9.62	

* p-value = Obtained by Fisher’s exact test

There was no association between pH values ​​and type of diet (p-value=0.4695). There was also no relationship between pH value and age, according to the Spearman correlation test of 0.17 (p-value = 0.327)^(^ 22 ^-^ 23 ^)^.

Radiological images showed that 160 newborns (98.77%) had the tube correctly placed in the body, bottom or antrum of the stomach. Among those incorrectly placed, one case was in the cardia and the other in the pylorus. Tube placement in the esophagus, duodenum, larynx or in the lower airways were not observed.

The accuracy of the pH test to verify the positioning of the tube had a sensitivity of 96.25% and specificity of 50% when compared to the results of radiological images. Positive predictive value, negative predictive value responses, and confidence intervals for all analyzes are presented in Table 2.

**Table 2:** Accuracy of the pH test to verify the placement of the gastric tube (n=162). Campinas, SP, Brazil, 2017

	%	CI*95%		Radiological images		
Sensitivity	96.25	(92.02; 98.61)	PH value	Correct	Incorrect	Total
Epecificity	50.00	(1.26; 98.74)	≤5.5	154	1	155
PPV†	99.35	(97.47; 99.84)	>5.5	6	1	7
NPV‡	14.29	(3.28; 45.04)	Total	160	2	162

* CI = Confidence Interval;

† PPV = Positive predictive value;

‡ NPV = Negative predictive value

Considering the result of the association between the use of histamine H_2_-receptor blockers and pH values > 5.5 of the aspirated gastric secretion, although the sample is small, sensitivity, specificity, positive predictive value and negative predictive factor alone in the 156 patients who did not receive this drug were also verified. It was observed that, in the evaluation of patients without gastric secretion inhibitor, sensitivity (98.70%) and positive predictive value (99.35%) remained high and there was no improvement in specificity (50.5%). However, there was an increase in the negative predictive value (33.33%), as shown in Table 3 with their respective confidence intervals.

**Table 3:** Accuracy of the pH test to verify the placement of the gastric tube in patients who did not receive histamine H_2_ receptor blockers (n=156). Campinas, SP, Brazil, 2017

	%	CI*95%		Radiological images		
Sensitivity	98.70	(95.39; 99.84)	PH value	Correct	Incorrect	Total
Epecificity	50.00	(1.26; 98.74)	≤5.5	152	1	153
PPV†	99.35	(97.44; 99.84)	>5.5	2	1	3
NPV‡	33.33	(6.62; 77.91)	Total	154	2	156

* IC = Confidence Interval;

† PPV = Positive predictive value;

‡ NPV = Negative predictive value

## Discussion

In the sample under study, we found that the mean pH of the gastric aspirate was, according to the literature, <4.0^(^ 16 ^)^. However, no relationship was observed between pH value and age. In addition, fasting or not and the type of diet did not influence the pH, considering the cutoff point specified for the proposed objective.

In contrast, a study that investigated the pH of gastric secretion in 96 newborns, according to the feeding pattern, found that the average pH of the subjects who were fed every 1 hour was higher than those fed every three hours (5.0; 3.5; p-value=0.001). Despite this difference, this result did not interfere with the evaluation of gastric tube placement, as both averages were below 5.0^(^ 16 ^)^.

Another factor that may influence the pH of gastric secretion is the use of histamine H_2_ receptor antagonist drug. Although its use is associated with an increased risk of infections and necrotizing enterocolitis, this drug is often used in neonatal care for stress ulcer prophylaxis and in the treatment of gastroesophageal reflux disease^(^ 24 ^-^ 25 ^)^.

In our sample, an association was observed between the use of the gastric secretion inhibitor drug and pH values> 5.5, but the number of subjects in which this occurred was small (n=4), and caution was required in evaluating these results. A study of 54 newborns^(^ 26 ^)^ found that the pH averages of subjects treated (4.89; SD 1.35) and untreated with gastric secretion inhibitors (3.43; SD 0.83). Despite the difference found (p-value=0.002), in both groups the mean was less than 5.5 and among the group of treated patients, 77% had pH values ​​≤5.5. In the study mentioned, no association analysis was performed between the use of gastric secretion inhibitors and pH values.

The sucrose solution administered minutes before insertion of the nasogastric tube had a pH value of 5.2, which was verified with a pH meter (744 pHmeter, Metrohm^®^, Switzerland). Because the solution was administered and absorbed orally, there was no influence on the result of the gastric pH test.

A cutoff point of 5.5 to determine the placement of the stomach tube in newborns is recommended by the UK National Patient Safety Agency and confirmed by other authors^(^ 10 ^,^ 26 ^)^, but in the literature we also find authors who recommend the cutoff point at 5.0^(^ 27 ^-^ 29 ^)^ and 6.0^(^ 16 ^.^ 30 ^)^.

In this study, the accuracy of the cutoff pH test showed a high sensitivity of 96.25%. When newborns treated with gastric secretion inhibitor were excluded from the analysis, sensitivity increased to 98.7%. The positive predictive value remained at 99.35% in both cases, revealing that there was no difference in the proportion of correctly positioned tubes among the tubes identified as positive by the pH test.

A recent study of 212 children^(^ 29 ^)^ investigated the pH of gastric secretion and endotracheal secretion in patients aged 3 days to 51 weeks and evaluated the accuracy of the pH test in distinguishing the two types of secretion with 4 different cutoff points: pH <4.0, <4.5, <5.0 and <5.5. In this study the subjects were divided into 4 groups, combining the use or not of a gastric secretion blocking drug with the presence or absence of recent feeding. Considering here only the recently fed subjects and the cutoff point <5.5, which is closest to our study, we observed similarity in the pattern of the results. In the study cited^(^ 29 ^)^, sensitivity was 96.1% and positive predictive value 98.0% in subjects treated with gastric secretion inhibitors, while sensitivity was 100% and positive predictive value 98.4% in those without using the drug.

It is noteworthy that, regarding the use of gastric secretion inhibitor drug, in our analysis two groups were not separated as in the study mentioned above^(^ 29 ^)^. The first analysis of our study refers to both treated and untreated patients with gastric secretion inhibitor, while the second analysis only to untreated patients.

When observing the results of specificity and negative predictive value, in our study we found low specificity (50%) in both analyzes. The negative predictive value was 14.29 in the general sample and 33.33 when excluding newborns treated with gastric secretion inhibitor, suggesting that the use of this drug seems to interfere with the number of false negative tests and with the proportion of true negatives. It is emphasized that the analysis of specificity and negative predictive value is directly influenced by prevalence, and in this case corresponds to the occurrence of incorrectly placement of tubes, which was in only 1.23% of the subjects.

In the study with 212 children^(^ 29 ^)^, considering only the analysis of the group of subjects recently fed with a cut-off point <5.5, a high specificity value (98.3%) was observed in the treated and non-treated with gastric acid secretion inhibitor subjects and negative predictive value of 96.7% among those receiving the drug and 100% among those not receiving.

The large discrepancy observed between the values ​​of specificity and negative predictive value presented here with the results of the mentioned study^(^ 29 ^)^ can be explained by the strategy adopted in the study cited, in which besides gastric secretion samples, samples were also collected from endotracheal tube secretion of 60 subjects (28.30% of the sample), which increased the occurrence of negative results and allowed to establish high specificity and negative predictive value.

Limitations of the present study include that findings related to the association of histamine H_2_-receptor blocker use and pH values> 5.5 may be inconclusive due to the small number of subjects in the sample receiving this drug. In addition, the occurrence of only two incorrectly placed tubes is directly related to the low specificity of the test. More substantial specificity could have been obtained if aspirate was collected from jejunal and / or endotracheal tube in the sample under study.

The findings are not yet generalizable, therefore, there is a need to replicate this study in neonatal patients with the same and different characteristics of the study sample, including patients with intubation, sedation and/or neurological disorders, with different gestational ages and patients with congenital malformations or syndromes.

## Conclusion

The use of pH reagent strips is a sensitive but non-specific test to verify the placement of the gastric tube in newborns in the sample studied. That is, pH values ​​≤5.5 in the aspirated gastric tube secretion are sensitive indicators of the correct positioning of the tip of the tube. However, pH values >5.5 were not specific for the incorrect placement of the tube.

In addition, there is evidence that the use of histamine H_2_ receptor antagonist drug may increase the pH value and cause confusion in the evaluation of gastric tube placement.

This study cooperates with the results found in the literature and suggests that, in neonatal patients with characteristics similar to the studied sample, pH reagent strips may be used as an adjuvant technique in the evaluation of gastric tube placement.

When interpreting the pH test results, values ​​≤5.5 point to correct gastric positioning, while values >5.5 would require radiological confirmation. Newborns on gastric acid secretion inhibitor drugs may have a false negative result, and radiological examination to confirm positioning is relevant.

## References

[1] Suryawanshi P, Dahat A, Nagpal R, Malshe N, Kalrao V (2014). A rare case of accidental esophageal perforation in an extremely low birth weight neonate. J Clin Diagn Res.

[2] Metheny NA, Meert KL (2014). A review of published case reports of inadvertent pulmonary placement of nasogastric tubes in children. J Pediatr Nurs.

[3] Farrington M, Lang S, Cullen L, Stewart S (2009). Nasogastric tube placement verification in pediatric and neonatal patients. Pediatr Nurs.

[4] Irving SY, Lyman B, Northington L, Bartlett JA, Kemper C (2014). Nasogastric tube placement and verification in children: review of the current literature. Crit Care Nurse.

[5] Clifford P, Heimall L, Brittingham L, Finn Davis K (2015). Following the evidence: enteral tube placement and verification in neonates and young children. J Perinat Neonatal Nurs.

[6] Dias FSB, Emidio SCD, Lopes MHBM, Shimo AKK, Beck ARM, Carmona EV (2017). Procedures for measuring and verifying gastric tube placement in newborns: an integrative review. Rev. Latono-Am. Enfermagem.

[7] Ellett MLC, Croffie JMB, Cohen MD, Perkins SM (2005). Gastric tube placement in young children. Clin Nurs Res.

[8] American Association of Critical-Care Nurses (AACN) (2016). AACN practice alert: initial and ongoing verification of feeding tube placement in adults.

[9] Fan E, Tan S, Ang S (2017). Nasogastric tube placement confirmation: where we are and where we should be heading. Proceedings of Singapore Healthcare (PoSH).

[10] National Patient Safety Agency (2005). Patient safety alert 09. Reducing the harm caused by misplaced naso and orogastric feeding tubes in babies under the care of neonatal units.

[11] Lyman B, Kemper C, Northington L, Yaworski JA, Wilder K, Moore C (2016). Use of temporary enteral access devices in hospitalized neonatal and pediatric patients in the united states. JPEN J Parenter Enteral Nutr.

[12] Child Health Patient Safety Organization, ECRI Institute (2012). Patient safety action alert. Event: blind pediatric ng tube placements - continue to cause harm.

[13] Parker L, Torrazza RM, Li Y, Talaga E, Shuster J, Neu J (2015). Aspiration and evaluation of gastric residuals in the neonatal intensive care unit: state of the science. J Perinat Neonatal Nurs.

[14] Powers J, Fischer MH, Ziemba-Davis M, Brown J, Phillips DM (2013). Elimination of radiographic confirmation for small-bowel feeding tubes in critical care. Am J Crit Care.

[15] Boyle JT (2003). Acid secretion from birth to adulthood. J Pediatr Gastroenterol Nutr.

[16] Freer Y, Lyon A (2005). Nasogastric tube aspirate pH values associated with typical enteral feeding patterns in infants admitted to an NICU. J Neonatal Nurs.

[17] Marciano T, Wershil BK (2007). The ontogeny and developmental physiology of gastric acid secretion. Curr Gastroenterol Rep.

[18] Pandey M, Datta V, Rehan HS (2013). Role of sucrose in reducing painful response to orogastric tube insertion in preterm neonates. Indian J Pediatr.

[19] Ziemer M, Carroll JS (1978). Infant gavage reconsidered. Am J Nurs.

[20] Freeman D, Saxton V, Holberton J (2012). A weight-based formula for the estimation of gastric tube insertion length in newborns. Adv Neonatal Care.

[21] Portney L, Watkins M (2009). Foundations of clinical research: applications to practice.

[22] Pagano M, Gauvreau K (2004). Princípios de bioestatística.

[23] Cohen J (1988). Statistical power analysis for the behavioral sciences.

[24] Terrin G, Passariello A, De Curtis M, Manguso F, Salvia G, Lega L (2012). Ranitidine is associated with infections, necrotizing enterocolitis, and fatal outcome in newborns. Pediatrics.

[25] Santana R, Santos V, Ribeiro R, Freire M, Menezes M, Cipolotti R (2017). Use of ranitidine is associated with infections in newborns hospitalized in a neonatal intensive care unit: a cohort study. BMC Infect Dis.

[26] Meert KL, Caverly M, Kelm LM, Metheny NA (2015). The pH of Feeding Tube Aspirates From Critically Ill Infants. Am J Crit Care.

[27] Gilbertson HR, Rogers EJ, Ukoumunne OC (2011). Determination of a practical pH cutoff level for reliable confirmation of nasogastric tube placement. JPEN J Parenter Enteral Nutr.

[28] Ellett ML, Cohen MD, Croffie JM, Lane KA, Austin JK, Perkins SM (2014). Comparing bedside methods of determining placement of gastric tubes in children. J Spec Pediatr Nurs.

[29] Metheny N, Pawluszka A, Lulic M, Hinyard L, Meert K (2017). Testing placement of gastric feeding tubes in infants. Am J Crit Care.

[30] Westhus N (2004). Methods to test feeding tube placement in children. MCN Am J Matern Child Nurs.

